# Distinct and content-specific neural representations of self- and other-produced actions in joint piano performance

**DOI:** 10.3389/fnhum.2025.1543131

**Published:** 2025-03-12

**Authors:** Natalie Kohler, Anna M. Czepiel, Örjan de Manzano, Giacomo Novembre, Peter E. Keller, Arno Villringer, Daniela Sammler

**Affiliations:** ^1^Department of Neurology, Max Planck Institute for Human Cognitive and Brain Sciences, Leipzig, Germany; ^2^Research Group Neurocognition of Music and Language, Max Planck Institute for Empirical Aesthetics, Frankfurt am Main, Germany; ^3^Department of Music, Max Planck Institute for Empirical Aesthetics, Frankfurt am Main, Germany; ^4^Department of Psychology, University of Toronto Mississauga, Mississauga, ON, Canada; ^5^Department of Cognitive Neuropsychology, Max Planck Institute for Empirical Aesthetics, Frankfurt am Main, Germany; ^6^Department of Neuroscience, Karolinska Institutet, Stockholm, Sweden; ^7^Neuroscience of Perception and Action Laboratory, Italian Institute of Technology, Rome, Italy; ^8^Center for Music in the Brain, Department of Clinical Medicine, Aarhus University, Aarhus, Denmark; ^9^The MARCS Institute for Brain, Behaviour and Development, Western Sydney University, Penrith, NSW, Australia; ^10^Department of Neuropsychology, Max Planck Institute for Human Cognitive and Brain Sciences, Leipzig, Germany

**Keywords:** joint action, fMRI, MVPA, music performance, pianists, motor simulation, internal models

## Abstract

During ensemble performance, musicians predict their own and their partners’ action outcomes to smoothly coordinate in real time. The neural auditory-motor system is thought to contribute to these predictions by running internal forward models that simulate self- and other-produced actions slightly ahead of time. What remains elusive, however, is whether and how own and partner actions can be represented *simultaneously* and *distinctively* in the sensorimotor system, and whether these representations are *content-specific*. Here, we applied multivariate pattern analysis (MVPA) to functional magnetic resonance imaging (fMRI) data of duetting pianists to dissociate the neural representation of self- and other-produced actions during synchronous joint music performance. Expert pianists played familiar right-hand melodies in a 3 T MR-scanner, in duet with a partner who played the corresponding left-hand basslines in an adjacent room. In half of the pieces, pianists were motorically familiar (or unfamiliar) with their partner’s left-hand part. MVPA was applied in primary motor and premotor cortices (M1, PMC), cerebellum, and planum temporale of both hemispheres to classify which piece was performed. Classification accuracies were higher in left than right M1, reflecting the content-specific neural representation of self-produced right-hand melodies. Notably, PMC showed the opposite lateralization, with higher accuracies in the right than left hemisphere, likely reflecting the content-specific neural representation of other-produced left-hand basslines. Direct physiological support for the representational alignment of partners’ M1 and PMC should be gained in future studies using novel tools like interbrain representational similarity analyses. Surprisingly, motor representations in PMC were similarly precise irrespective of familiarity with the partner’s part. This suggests that expert pianists may generalize contents of familiar actions to unfamiliar pieces with similar musical structure, based on the auditory perception of the partner’s part. Overall, these findings support the notion of parallel, distinct, and content-specific self and other internal forward models that are integrated within cortico-cerebellar auditory-motor networks to support smooth coordination in musical ensemble performance and possibly other forms of social interaction.

## 1 Introduction

Coordinating own actions with the actions of a partner is necessary in many kinds of situations, such as holding a conversation, playing soccer or performing music in groups. One key component of successful interaction is the ability to predict the partner’s action ahead of time to swiftly adapt one’s own action if needed (Abalde et al., 2024; Knoblich et al., 2011; Vesper et al., 2017). It has been argued that these predictions can be formed via motor simulation of the partner action in one’s own motor system (Kilner, 2011; Ridderinkhof, 2014; Sebanz et al., 2006; Wilson and Knoblich, 2005; Wolpert et al., 2003). However, if action coordination indeed involves the “motoric embodiment” of the partner, it remains elusive whether and how self- and other-produced actions are represented *simultaneously* and *distinctively* in the motor system during joint action. Moreover, many studies have focused primarily on global activity changes as proxy for predictive motor simulation (Bolt and Loehr, 2021; Calvo-Merino et al., 2006; Kohler et al., 2023), leaving unclear whether the motor system has distinct representation of the *specific content* of the partner’s action. The present study capitalized on an existing functional magnetic resonance imaging (fMRI) dataset of duetting pianists (Kohler et al., 2023) to fill these gaps by seeking to dissociate neural representations of self- and other-produced actions during synchronous joint music performance using multivariate pattern analysis (MVPA).

### 1.1 The motor system in individual and joint action

Coordination of social interactions often benefits from knowing what others will do next. While there are numerous ways of predicting others’ actions, e.g., based on abstract action schemas (Sartori et al., 2011, 2013; Wurm and Schubotz, 2017) or representations of action goals in space and time (Sebanz et al., 2006; Sebanz and Knoblich, 2009; Vesper et al., 2010), one mechanism that has been most central in theories of joint action is the simulation (sometimes called emulation or co-representation) of the partner action in one’s own motor system (Hommel, 2009; Knoblich and Sebanz, 2006; Koch et al., 2010; Ridderinkhof, 2014; Sebanz et al., 2006; Vesper et al., 2010). Originally inspired by James (1890) ideomotor principle and based on the social “extrapolation” of motor control theories of self-produced actions (Miall and Wolpert, 1996; Wolpert et al., 2003), these (simulation) theories assume that we anticipate the outcome of partner actions very much in the same way as we anticipate the sensory consequences of our own actions: by running internal forward models in our sensorimotor system (Keller et al., 2007, 2016; Müller et al., 2021; Novembre and Keller, 2014; Patel and Iversen, 2014).

Internal forward models—originally studied in the context of self-produced actions—transform motor commands into a prediction of the sensory consequences of a movement (for review, see Ishikawa et al., 2016). These models are based on stored sensorimotor associations that are acquired during practice of the corresponding action and increase in precision with training (Jeannerod, 2006; Wolpert et al., 2011). In terms of neural correlates, internal forward models have been associated with cortico-cerebellar loops. Accordingly, the cerebellum integrates the efference copy of the ongoing motor command issued in primary and premotor cortex (M1/PMC), and afferent sensory signals from the periphery. Based on learned sensorimotor links, the cerebellum estimates future sensory input, evaluates the accuracy of this estimation given the actual input, and links back to M1/PMC in case of a mismatch to adapt the movement (for reviews, see Bastian, 2006; Ishikawa et al., 2016; Ito, 2005; Johnson et al., 2019; Popa and Ebner, 2019; Tanaka et al., 2020; Wolpert et al., 1998). Importantly, this cortico-cerebellar “pre-play” or simulation of the action allows the sensorimotor system to preemptively detect (and potentially avert) impending execution errors in self-produced actions (Maidhof, 2013; Maidhof et al., 2009; Ruiz et al., 2009).

Evidence from action observation studies suggests that the outcome of *other*-produced actions is anticipated similarly in an agent’s motor system, to seamlessly adapt to the behavior of interaction partners (Pacherie, 2008). For example, the cortical motor system, including PMC and inferior/superior parietal lobule (IPL/SPL), is robustly activated during action observation (for reviews, see Caspers et al., 2010; Hardwick et al., 2018; Papitto et al., 2020) taken to reflect motor simulation. Importantly, motor activity increases with the (motoric) familiarity of the observed actions (e.g., Calvo-Merino et al., 2005, 2006; Kohler et al., 2023; Ticini et al., 2019; Tomeo et al., 2013), often maps onto the somatotopy of the observed body kinematics, and—crucially—facilitates the anticipation of observed action outcomes (e.g., Aglioti et al., 2008; Candidi et al., 2014; Urgesi et al., 2012). This is in line with the idea that motor simulation of others’ actions is predictive, and based on specific, practice-based sensorimotor associations, like internal forward models of self-produced actions.

Interestingly, motor activity associated with observed or real partner actions is stronger in interactive than non-interactive or solo contexts (e.g., Novembre et al., 2012; Sacheli et al., 2022; for review, see Bolt and Loehr, 2021). This activity increase in joint action may reflect a more detailed and exact simulation of a (potential) partner’s action, leading to more accurate predictions that serve to smoothen coordination. If so, this would not only provide evidence that shared goals and task interactivity shape the use of motor simulation (see also Sacheli et al., 2019), but also highlight the need to investigate the neural processes underlying joint action in real social interactive settings (Redcay and Schilbach, 2019; Schilbach et al., 2013).

A number of studies have answered this call for interactive settings using musical joint action tasks. These studies typically asked pianists to perform duets with a (real or videotaped) partner, whereby one pianist played the right-hand melody and the other the left-hand bassline (c.f. Novembre et al., 2012). The critical manipulation was familiarity, that is, whether—prior to the experiment—pianists had or had not practiced the partner’s part. If internal forward models depend on learned auditory-motor associations acquired during practice, predictive motor simulation should be stronger and more accurate during pieces with familiar compared to unfamiliar partner actions, and should have measurable behavioral effects on interpersonal coordination. Indeed, the fMRI study of Kohler et al. (2023) found stronger cortico-cerebellar activity (including M1, PMC, and cerebellar lobule VIII), stronger auditory-motor connectivity, and greater cerebellar sensitivity to subtle temporal asynchronies when pianists were familiar than unfamiliar with the other’s part. Correspondingly, inhibitory transcranial magnetic stimulation (TMS) of right M1/PMC (controlling the left hand, used by the partner) was found to perturb the temporal accuracy of pianists’ own right-hand entries when taking turns in duets (Hadley et al., 2015) and to reduce pianists’ precision in adapting to tempo changes induced by the duet partner (Novembre et al., 2014), but only when pianists were familiar with the partner’s (left-hand) part. These combined results (see also Novembre et al., 2016; Ragert et al., 2013) support the assumption that internal forward models of familiar partner actions may be more accurate and boost the anticipation of an action’s time course, with consequences for the temporal coordination of joint action.

However, what remains unclear is whether the motor system represents the *specific content* of the partner’s action, and how it does so *simultaneously* with the execution of one’s own action. So far, both TMS and fMRI evidence mainly builds on global activity changes, leaving unclear whether self and partner representations are really content-specific. How veridically do they reflect the kinematics of own and partner actions? Some TMS studies provide suggestive evidence for content-specificity by showing muscle-specific changes of cortico-spinal excitability that mirror complementary self- and other-produced actions observed in videos (Sartori et al., 2013, 2015). However, findings from other studies probing muscle-specific effects of partner actions in real synchronous musical interactions were not conclusive (Novembre et al., 2012; Novembre and Keller, 2014). More generally, it is rather difficult to test simultaneous self- and other-related representations in real interactive settings while measuring cortico-spinal excitability. An alternative approach to study action specificity of neural representations in joint action is to combine neuroimaging (fMRI) with multivariate pattern analysis (MVPA). In contrast to the coarseness of univariate measures that rely on global activity differences, MVPA capitalizes on information contained within fine-scale spatial activation patterns. If neural representations of partner actions are content-specific (e.g., reflecting a particular finger sequence), they should evoke specific patterns of activity across fMRI voxels, from which individual actions or action sequences may be decoded (Peelen and Downing, 2023). We applied MVPA to the fMRI dataset of Kohler et al. (2023) to investigate on this fine-grained level whether and how the motor system concurrently represents self- and other-produced actions during synchronous joint music performance.

### 1.2 Decoding own actions

Previous fMRI studies using MVPA have shown that the execution as well as motor imagery of self-produced hand actions is reflected in action-specific neural representations in the motor system. For example, simple actions like reaching vs. grasping (Gallivan et al., 2011, 2013; Gallivan and Culham, 2015), different types of grasps (Michalowski et al., 2022; Turella et al., 2013), and complex finger-movement sequences (Kornysheva and Diedrichsen, 2014; Wiestler et al., 2014; Wiestler and Diedrichsen, 2013) could be accurately classified based on patterns of brain activity. Crucially, accurate classifications occurred in a broad range of sensorimotor regions, including M1, primary somatosensory cortices (S1), PMC, intraparietal sulcus (IPS), and the cerebellum. Neural activity patterns in similar sets of regions, including M1, S1, PMC and additional visual cortices, have also been found to represent *imagined* actions, such as simple reaching (Filimon et al., 2015), pointing and squeezing actions (Pilgramm et al., 2016; Zabicki et al., 2016, 2019) or different types of grasps (Monaco et al., 2020), as well as complex whole-body actions (Yang et al., 2023). Most importantly, the neural representations of own, unimanual actions are often strongly lateralized. For example, neural activity patterns representing (sequences of) right-hand finger movements were found to be more distinctive in left than right M1/PMC, i.e., contralateral to action execution (Wiestler et al., 2014; Yokoi et al., 2018), although lateralization is sometimes less clear-cut in PMC (Michalowski et al., 2022). Moreover, neural representations of finger sequences become more refined after practice, i.e., classification accuracy increases with motor familiarity (Wiestler and Diedrichsen, 2013), in line with the idea that content-specific motor representations are shaped by training.

Motor familiarity with an action has also been shown to strengthen expectations of the sensory consequences of that action, e.g., sounds during music production (Baumann et al., 2007; Jäncke, 2012; for review, see Zatorre et al., 2007), in line with the assumption that internal forward models are built on learned sensorimotor associations. For example, pianists exhibited stronger ERP responses when perceiving errors in auditory melodies that belonged to their motor repertoire compared to unrehearsed melodies (Mathias et al., 2015), and pianists’ sensitivity to altered auditory feedback during own performance increased with the amount of musical training, in line with increasing precision of internal forward models with training (Pfordresher, 2012). More generally, previous MVPA studies showed content-specific neural activity patterns for perceived and/or imagined musical melodies (de Manzano et al., 2020; May et al., 2022; Regev et al., 2021; Schindler et al., 2013) in the superior temporal gyrus (STG), including Heschl’s Gyrus (HG) and planum temporale (PT). Importantly, these auditory representations were more precise not only in highly trained listeners with more differentiated tonal knowledge (May et al., 2022), but also when listeners tapped along (Regev et al., 2021) or had previously practiced the heard melodies (de Manzano et al., 2020) in line with strengthened auditory representations through auditory-motor coupling (Kohler et al., 2023).

Taken together, execution and imagery of self-produced actions are reflected in action-specific neural activity patterns in the motor system. These activity patterns, especially in M1, are lateralized, increase in precision with motor familiarity, and are associated with auditory representations. Both the lateralization and the training-induced refinement of neural action representations may provide us with a means to dissociate representations of self- and other-performed actions in the present study, as explained below.

### 1.3 Decoding others’ actions

Increasingly, MVPA studies focus on action observation (for review, see Oosterhof et al., 2013). These studies collectively demonstrate highly specific representations of others’ actions in the observer’s brain, in terms of movement kinematics (Ridderinkhof et al., 2021; Ziccarelli et al., 2022), action goals (e.g., Molenberghs et al., 2012), or even abstract intentions (e.g., Koul et al., 2018). Typically, observing other-produced actions yields neural representations in similar motor regions as self-produced actions. For example, simple reaching actions (Filimon et al., 2015), different types of grasps (Errante et al., 2021; Sacheli et al., 2019), (non)social and (in)transitive hand actions (Lesourd et al., 2023), as well as complex finger sequences (Apšvalka et al., 2018) presented in videos have been reliably classified based on activity patters in areas including left PMC, inferior/superior parietal lobule (IPL/SPL), and the right cerebellum (lobule VI and VIII), known to support own (right-hand) action execution (see above). Notably, classification of observed actions in PMC was more accurate in social interactive, compared to non-interactive, contexts (Sacheli et al., 2019) in line with the idea that sharing a goal with a co-actor shapes the accuracy of motor simulation and representations (Sacheli et al., 2022).

Overall, this research suggests that both self- and other-produced actions evoke action-specific patterns of brain activity in the motor system.

### 1.4 Current study and predictions

In the current study, we investigated how self- and other-produced actions are represented *simultaneously* in the sensorimotor system during synchronous joint action. To test this, we reanalyzed data of a previous study, in which pairs of pianists performed duets together (Kohler et al., 2023). One pianist played the right-hand part (melody) of the duets in an MR-scanner, while the co-performer played the corresponding left-hand part (bassline) on a piano outside the scanner room. To investigate whether and how pianists (in the scanner) neurally represent the left-hand actions of the co-performer, on top of their own right-hand actions, we manipulated their motor familiarity with the part played by the co-performer. That is, for half of the pieces performed in the MR-scanner (*N* = 2), pianists had practiced the co-performer’s part (the bassline) prior to the experiment, while they had neither practiced, nor heard or seen the scores of their partner’s basslines for the other half of the pieces (*N* = 2). Hence, they were motorically familiar or unfamiliar with their partner’s actions, respectively.

We used multivariate pattern analysis (MVPA) in auditory-motor regions of interest (ROIs) and the whole brain (searchlight) to dissociate neural representations of self-produced right- and other-produced left-hand actions. ROIs were localized in left and right primary motor and premotor cortices (M1 and PMC), cerebellar lobule VIII (referred to as CER), and planum temporale (PT) based on the results of Kohler et al. (2023), that is, covering relevant areas of action execution, motor simulation and auditory perception/anticipation as introduced above. More precisely, these regions had shown stronger activity or functional connectivity when the partner played familiar (compared to unfamiliar) basslines, taken to indicate that these regions represent not only own actions, but also the actions produced by the partner (Kohler et al., 2023). In each ROI (and searchlight), we ran two separate MVPAs classifying which of two pieces was performed, separately for the two pieces with familiar and the two pieces with unfamiliar partner actions. Classification accuracies of these two analyses were compared within each ROI (and searchlight), and between the left and right hemisphere.

Following established knowledge of lateralized motor control (Chettouf et al., 2020; Goble and Brown, 2008; Welniarz et al., 2015), we reasoned that classification accuracies in left M1/PMC and right CER are primarily associated with self-produced right-hand actions, while classification accuracies in right PMC and left CER are rather associated with other-produced left-hand actions. Crucially, if motor simulation in internal forward models depends on specific, practice-based sensorimotor familiarity, neural representations in right PMC and left CER should be more precise, i.e., classification accuracies should be higher, for pieces with motorically familiar than unfamiliar partner actions. Finally, we explored the possibility of a similar effect of familiarity in (bilateral) PT, under the assumption that internal forward models of other-produced actions trigger more precise auditory sequence representations (de Manzano et al., 2020; Kohler et al., 2023; Regev et al., 2021). However, we also considered it possible that the (top-down) influence of motor familiarity on auditory representations might be cancelled out by the actually perceived (bottom-up) auditory input.

## 2 Methods

The current study reanalyzed the data of Kohler et al. (2023). Key details of the experimental methods are outlined below (for further information, see Kohler et al., 2023).

### 2.1 Participants

Forty expert pianists (age range: 18–39 years, *M* = 25.25 years, *SD* = 5.30, 4 left-handed, 20 identified as female, 20 identified as male) with an average of 17.18 years of piano training (*SD* = 5.86, range: 8–32 years; onset age *M* = 7.70, *SD* = 3.07, range: 4–16 years) and an average of 8.73 h of weekly practice at the time of testing (*SD* = 9.69, range: 2–50 h) were randomly allocated into 20 pairs (4 only-female, 4 only-male, 12 mixed-gender pairs, mean age difference between partners: 5.30 years, *SD* = 4.43). Pianists did not know each other before the experiment. Handedness of the pianists was assessed using the Edinburgh Handedness Inventory (Oldfield, 1971). All pianists had normal or corrected-to-normal vision, reported normal hearing, no neurological or psychiatric history, and no contraindication for MRI. They were naïve to the purpose of the study and received monetary compensation for their participation. The study was approved by the ethics committee of Leipzig University (016–15-26012015) and was conducted following the guidelines of the Declaration of Helsinki. All pianists provided written informed consent.

### 2.2 Materials

**Figure 1:**
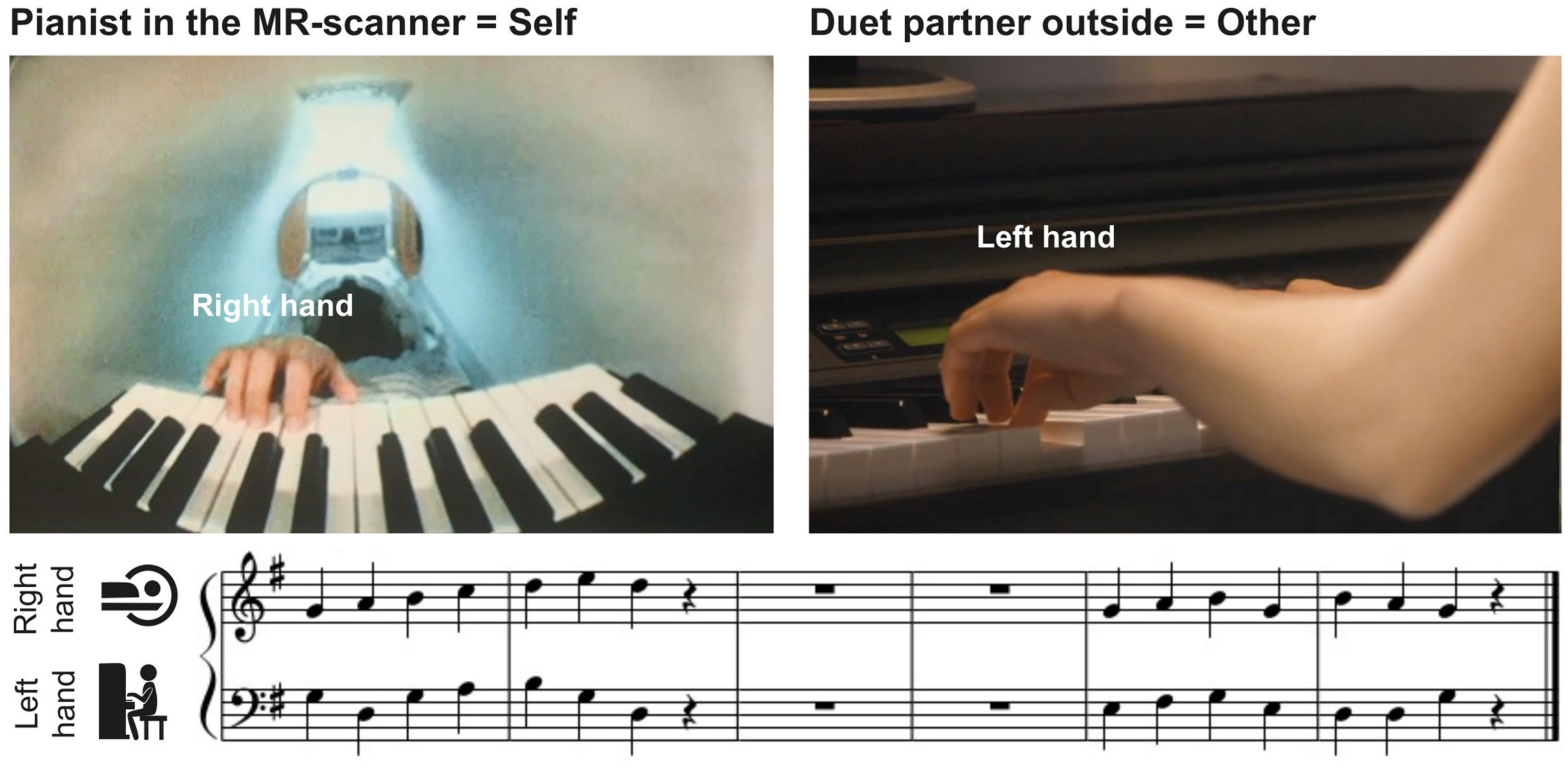
Experimental setup. Pianists in the MR-scanner (self, left upper panel) performed right-hand melodies in duet with a partner (other, right upper panel) who played the corresponding left-hand basslines outside the scanner room. Pianists saw the musical scores of their own, but not the partner’s part, on a screen (see lower panel for an example).

The musical material consisted of 8 excerpts of modified chorales by Johann Sebastian Bach, with a melody for the right hand played by the pianist in the scanner and a bassline for the left hand played by the duet partner outside (see Figure 1 for an example). Each chorale contained one musical phrase of 2 bars, a pause of 2 bars, followed by another musical phrase of 2 bars. Each musical phrase consisted of 7 quarter notes and a quarter-note pause. The 8 excerpts were split into 2 sets of 4 pieces, which were used when player A or player B of a pair were in the MR-scanner, respectively (see below).

Approximately 2 weeks prior to the experiment, pianists received the scores of both sets of pieces for rehearsal at home. Crucially, to manipulate motor familiarity with the partner’s part, pianists received full scores for only half of the pieces (2 in each set), for which they were asked to practice both their own and their partner’s part (melody and bassline, respectively). These pieces were hence those with *familiar* (F) partner actions. For the remaining pieces, pianists received partial scores of only one part, i.e., they could practice either only the melody (2 pieces of the set they later performed inside the MR-scanner) or only the bassline (2 pieces of the other set). These pieces were hence those with *unfamiliar* (U) partner actions. The pieces for which both parts were practiced were counterbalanced across the group. Only pianists who were able to perform the practiced parts by heart in a pre-test were admitted to the experiment (for details, see Kohler et al., 2023).

An additional manipulation in the original study design of Kohler et al. (2023) required pianists to perform a tempo change in the second phrase (i.e., after the pause) which was executed without auditory feedback. The present analysis focused on the first phrase only (i.e., before the pause when pianists could hear each other) to study auditory-motor representations of self and other. A control analysis confirmed that the tempo manipulation in the second phrase had no effect on the present results in the first phrase (Supplementary Table S2).

### 2.3 Experimental procedure

The fMRI experiment consisted of 2 consecutive scanning sessions separated by a 30-min break. A short training (16 trials) at the beginning of each session ensured that pianists had understood the instructions, were able to play the rehearsed pieces together, and heard each other’s performance well via headphones. In the first session, pianist A played the piano in the MR-scanner in duet with co-performer B who played outside the scanner room. They swapped places in the second session. The pianist in the MR-scanner always played the melody of the pieces with the right hand, while the co-performer played the corresponding bassline with the left hand (Figure 1). During each session, the pianists played a set of 4 of the 8 practiced pieces, counterbalanced across pairs. They completed 128 trials in each session, 64 with familiar and 64 with unfamiliar partner actions, in pseudorandom order such that partner actions were familiar or unfamiliar in not more than three consecutive trials, and the same piece was never played twice in a row. Each piece was played 32 times over the course of the session.

The first phrase in each trial was played at a tempo of 120 bpm, while the second phrase had to be performed either at 150 bpm (faster) or 96 bpm (slower). Note that only the first phrase without tempo change was analyzed in the current study (for details on the tempo manipulation, see Kohler et al., 2023). Each trial started with a visual cue (1,000 ms) that indicated whether to speed up or slow down in the second phrase. After the cue, the musical scores of the pianist’s respective part (but not the partner’s part) appeared on screen and four metronome beats were presented at a tempo of 120 bpm (lasting 2,000 ms in total) after which pianists were supposed to start playing together at that same tempo. Trials lasted between 14.2 s and 16 s, depending on the tempo of the second phrase. The next trial started after a jittered inter-trial-interval between 3 and 9 s during which a fixation cross was shown. One fMRI scanning session lasted about 45 min. The whole experiment, including preparation time, two sessions and breaks, took about five hours per pair.

### 2.4 Experimental setup and data acquisition

In the scanner, behavioral data were acquired via a custom-made 27-key MR-compatible MIDI-piano (Julius Blüthner Pianofortefabrik GmbH, Leipzig, Germany; see Figure 1), with auditory feedback received via MR-compatible in-ear headphones (Sensimetrics, MR confon GmbH, Magdeburg, Germany). The piano was placed on a slightly tilted wooden stand clipped into the scanner bed over the pianist’s lap. An MR-compatible camera (12 M camera, MRC Systems, Heidelberg, Germany) was placed on top of the piano to record the pianist’s finger movements. A double mirror system mounted on the head coil allowed the pianist to see both the piano and the visual stimuli projected onto a screen at the head-end of the MR-scanner. Pianist B was seated in a separate room at a Yamaha Clavinova CLP 150 on top of which a 16” Sony Trinitron Multiscan E220 monitor (100-Hz refresh rate) was placed for presentation of visual stimuli. Sound was delivered via DT 770 PRO, 250 Ohms headphones (beyerdynamic, Heilbronn, Germany). The audio-output of both pianos was fed into and mixed through an McCrypt SA-101 U USB DJ-mixer (Renkforce, Conrad Electronic SE, Hirschau, Germany) that was located in the control room where the experimenters were seated. The experiment was controlled with Presentation software (Version 16.5, Neurobehavioral Systems, Inc., Berkeley, CA, United States) and custom Python programs to record the MIDI output of the pianos.

MR-data were collected at the Max Planck Institute for Human Cognitive and Brain Sciences, Leipzig, in a 3-Tesla Siemens Skyra magnetic resonance scanner (Siemens AG, Erlangen, Germany) using a 32-channel head coil. Functional images were acquired with a whole-brain multi-band echo-planar imaging sequence (EPI; TR = 2,000 ms, TE = 22 ms, multi-band acceleration factor = 3, 60 axial slices in interleaved order, voxel size = 2.5 mm^3^, 10% inter-slice gap, flip angle = 80°, field of view = 204 mm; Feinberg, 2010; Moeller et al., 2010). Anatomical T1-weighted images were acquired with a whole-brain magnetization-prepared rapid acquisition gradient echo sequence (MPRAGE; TR = 2,300 ms, TE = 5.52 ms, 176 sagittal slices, voxel size = 1 mm^3^, flip angle = 9°, field of view = 256 mm; Mugler and Brookeman, 1991).

### 2.5 FMRI data analysis

To evaluate how self- and other-produced actions are neurally represented during joint music performance, we used MVPA to decode, in predefined ROIs, which piece pianists performed. Decoding was done separately for the two pieces with familiar and with unfamiliar partner actions, in bilateral M1, PMC, CER, and PT. Classification accuracies were then statistically compared between (un)familiar pieces and homologous left and right hemispheric ROIs using repeated measures ANOVAs. An analogous whole-brain searchlight MVPA was applied to explore potential representations of self- and other-produced actions outside the predefined auditory-motor ROIs.

#### 2.5.1 Preprocessing

FMRI data were pre-processed using SPM12 (Wellcome Trust Centre for Neuroimaging, London, UK) in Matlab version 9.3 (R2017b). Preprocessing included slice-time correction, realignment, unwarping, and co-registration of functional and anatomical scans, as well as segmentation.

#### 2.5.2 First-level design matrix

To build individual level design matrices, trials were first grouped into four predictors, i.e., one predictor for each piece depending on whether pianists were (un)familiar with their partner’s part. Predictors were labelled familiar piece 1 (F1), familiar piece 2 (F2), unfamiliar piece 1 (U1), and unfamiliar piece 2 (U2). Each predictor was then split into 8 folds across time to simulate separate runs and allow training and testing of the classifier including cross-validation. Each fold contained 4 trials of the respective piece, except for two participants for whom we included only 2–3 or 3–4 trials in each fold due to early termination of the session. The resulting 32 predictors were labelled by piece (F1, F2, U1, U2) and numbered 1–8, respectively. Furthermore, 6 motion parameters were entered as covariates of no interest to control for subtle head movements.

We modelled brain activity using a General Linear Model with finite impulse response (FIR) functions at a lag of +4 s relative to trial onset to account for the lag of the hemodynamic response. We used a FIR model rather than a canonical hemodynamic response model (HRF) to isolate brain activity specifically during pianists’ joint performance during the first phrase, and to avoid blurring this stage with activity of the adjacent stages of the trial. The FIR model was composed of 4 separate impulse functions with a length of 4 s each, modelling the 4 consecutive stages within trials, resulting in 4 beta images for each piece and fold. The first beta image reflected brain activity associated with the presentation of the visual cue and scores and hearing the metronome. Beta image 2 reflected activity evoked by the joint performance during the first phrase and was relevant for the present analysis. Beta images 3 and 4 reflected the pause and the subsequent second phrase, respectively. Only beta image 2 data were used in the MVPA. The final design matrix of each participant consisted of 134 columns, comprising 4 pieces (F1, F2, U1, U1) × 8 folds (with ~4 trials of each piece) × 4 functions of the FIR model +6 motion parameters.

#### 2.5.3 Definition of grey matter masks

All analyses were confined to grey matter voxels. Therefore, a structural grey matter mask was created in native-space for each participant, following the pipeline of de Manzano et al. (2020). First, individual grey matter tissue probability maps obtained during segmentation were thresholded at 0.5, then smoothed by 6 mm FWHM, and thresholded again at 0.2. The resulting images were then re-sliced to match the functional masks generated by SPM during the first-level analysis. Only voxels contained in both the functional masks and the grey matter maps were retained in the final native-space grey matter masks for individual-level analyses. For the group-level searchlight analysis, a group-level grey matter mask was created by normalizing all native-space grey matter masks into MNI space and retaining only voxels common to all individual masks.

#### 2.5.4 Definition of regions of interest (ROIs)

**Figure 2:**
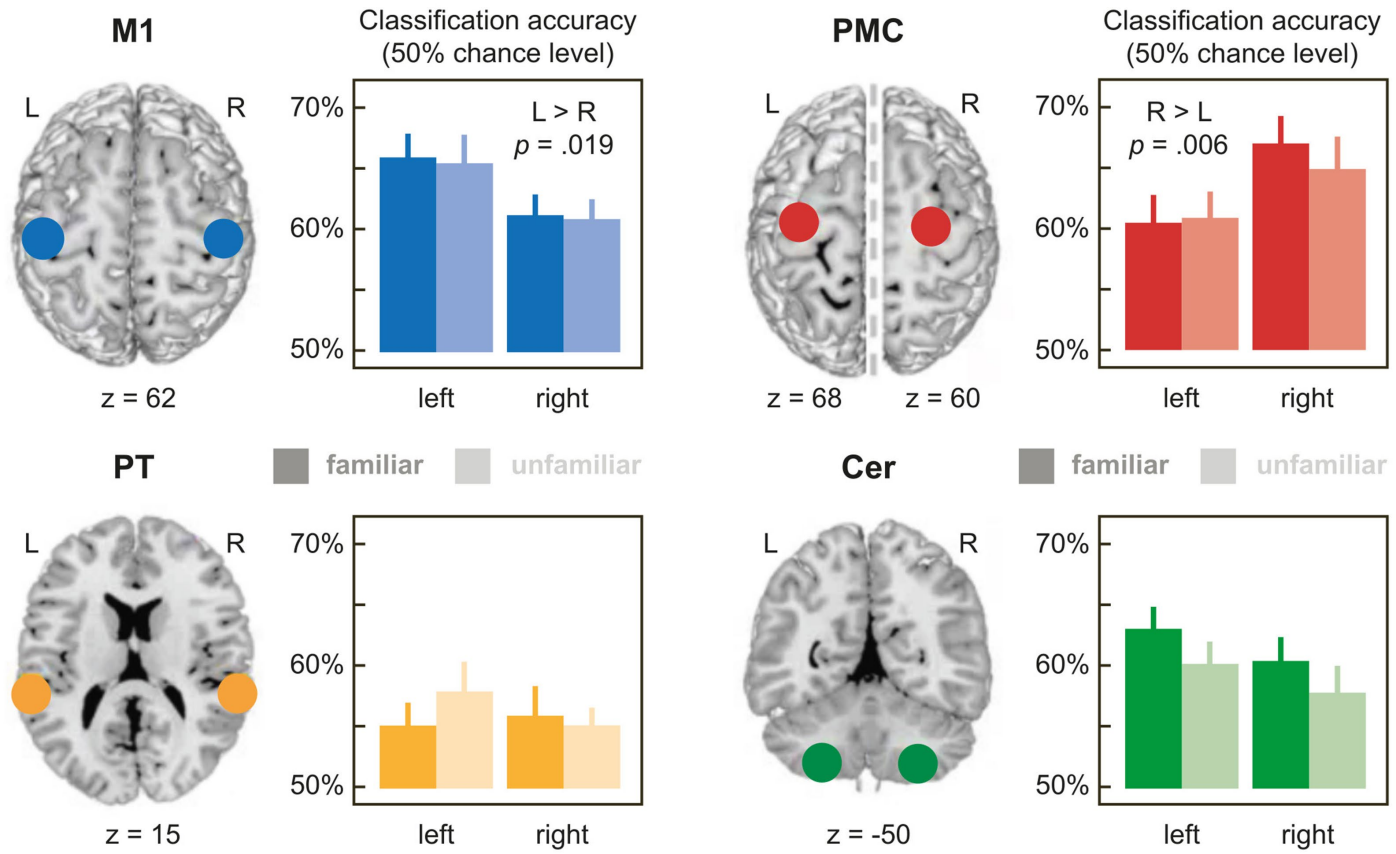
Mean accuracies for the classification of pieces with familiar (dark bars) and unfamiliar partner actions (light bars) in the 4 bilateral ROIs with 6 mm radius (for a full list of accuracies in the ROIs with 4 mm, 6 mm or 8 mm radius, see Supplementary Table S1). 50% on the y-axis corresponds to empirical chance level. M1: primary hand motor cortex; PMC: premotor cortex; PT, planum temporale; CER, lobule VIII of the cerebellum. Error bars denote ±1 *SEM*.

MVPA was first conducted in predefined ROIs in bilateral M1, PMC, CER, and PT, i.e., auditory-motor regions involved in joint action. More specifically, in Kohler et al. (2023), these regions had shown stronger activity or functional connectivity when pianists performed duets with a partner who played familiar (compared to unfamiliar) basslines. Given that motor simulation in internal forward models depends on motor familiarity (e.g., Calvo-Merino et al., 2005, 2006; Ticini et al., 2019; Tomeo et al., 2013; Jeannerod, 2006; Wolpert et al., 2011), we considered these areas as most promising candidates for representing partner actions, on top of own actions. ROIs were created using the MarsBaR toolbox for SPM12 (Brett et al., 2002) by centering spheres on MNI group coordinates obtained in Kohler et al. (2023). Right PMC [26–12 60], left PMC [−32–10 68], and left M1 [−44–22 62] corresponded to peak coordinates of clusters showing stronger activity when performing pieces with familiar compared to unfamiliar partner actions (see the univariate contrast in Kohler et al., 2023). To obtain coordinates for right M1 [44–22 62], the sign of the left M1 x-coordinate was flipped. Both M1 coordinates were located in the primary hand motor area reported in a meta-analysis by Mayka et al. (2006). The ROI in left cerebellar lobule VIII (CER) [−26–56 -50] was centered on the peak coordinates of a cluster that had shown sensitivity to subtle temporal asynchronies between pianists’ keystrokes when they were familiar (compared to unfamiliar) with their partner’s actions (see Kohler et al., 2023). The homologous right CER coordinates [26–56 -50] were again obtained by flipping the sign of the x-coordinate. According to the Cerebellar atlas (Diedrichsen et al., 2009) of the SPM Anatomy Toolbox (Eickhoff et al., 2005, 2006, 2007), these coordinates lay in lobule VIII with a probability of 76%. Finally, the center coordinates for left and right PT [±60–30 15] were selected based on their stronger functional connectivity with motor areas when pianists were familiar (compared to unfamiliar) with their partner’s part (see Kohler et al., 2023). Note that these coordinates were slightly shifted compared to Kohler et al. (2023), to increase the probability of assessing representations in PT. According to the Harvard-Oxford Cortical Structural Atlas (Desikan et al., 2006; Mazziotta et al., 2001), both coordinates lay in PT with probabilities of 49% for the right, and 52% for the left hemisphere. ROI locations are visualized in Figure 2.

We built spheres with 4 mm (9 voxels), 6 mm (33 voxels) and 8 mm radius (79 voxels) around each of these 8 coordinates. Three sphere sizes were used following the procedure of de Manzano et al. (2020) in order to control for ROI size (see recommendation by Shashidhara et al., 2020). The resulting 24 spheres (4 regions × 2 hemispheres × 3 sizes) were then transformed into native space by using the individual deformation fields obtained when normalizing individual brains to MNI standard space. Finally, the individual native space ROIs were reduced to grey matter voxels by performing a conjunction between the ROIs and the individual grey matter masks described above.

#### 2.5.5 Multivariate pattern analysis in regions of interest (ROI)

Multivariate pattern analyses were carried out in each ROI using the CoSMoMVPA toolbox (Oosterhof et al., 2016) in Matlab. First, the beta images corresponding to brain activity during the joint performance of the first phrase (see above) were demeaned to ensure that the results would not be merely driven by differences in activity strength. Then, a linear support vector machine (SVM) (Chang and Lin, 2011) was used to classify which of two pieces was performed, separately for the pieces with familiar (F1, F2) and for the pieces with unfamiliar (U1, U2) partner actions. We used a leave-2-out cross-validation scheme, i.e., trained the classifier on 6 folds and tested on the 2 remaining folds. Training and testing were done exhaustively on all 28 possible combinations of folds per subject. Classification accuracies of all 28 iterations were averaged, per participant and region. To estimate the individual chance level, the same procedure was repeated 10,000 times with randomly labelled trials for each participant and ROI. Chance level was found to be 50% in all cases (Supplementary Table S1). Paired *t*-tests with FDR-correction were used to ensure that classification accuracies were significantly higher than this empirical chance level.

Further statistical analyses were restricted to accuracies above chance, and were performed on the differences between accuracies and empirical chance-level, referred to as relative accuracies. Relative accuracies were compared in 3-way repeated measures ANOVAs with the factors FAMILIARITY (familiar, unfamiliar partner actions), HEMISPHERE (left, right), and SIZE of sphere radius (4 mm [only for M1, PMC], 6 mm, 8 mm), separately for each ROI (M1, PMC, CER, PT). All ANOVAs were performed using the *ez* package (Lawrence, 2016) in R.

#### 2.5.6 Multivariate pattern analysis with whole-brain searchlight approach

To explore whether any regions outside the predefined auditory-motor ROIs represent self- and other-produced action, we conducted a whole-brain searchlight MVPA analogous to the ROI-based analyses. The same SVM classifier and leave-2-out cross-validation scheme was used to classify pieces with familiar (F1, F2) and unfamiliar (U1, U2) partner actions in a searchlight moving through each participant’s grey matter mask (see above). The searchlight sphere had a 7.5 mm (3 voxel) diameter as suggested in previous studies (de Manzano et al., 2020; Kriegeskorte et al., 2006). For each participant, unfamiliar classification accuracy maps were subtracted from familiar maps, assuming similar chance-levels for both conditions (Supplementary Table S1).

The resulting difference maps were then normalized to MNI space and combined into a 4D volume, containing one 3D volume per participant. A one-sided one-sample *t*-test against zero was performed on this 4D MNI accuracy map using SPM 12, to identify regions in which classification accuracy was higher when partner actions were familiar compared to unfamiliar. To correct for multiple comparisons, threshold-free cluster-enhancement (Smith and Nichols, 2009) was applied through Monte Carlo simulation (Oosterhof et al., 2016) with a threshold of *α* = 0.05.

## 3 Results

### 3.1 Multivariate pattern analysis in regions of interest (ROI)

Familiar and unfamiliar pieces were classified significantly above empirical chance level in all M1 and PMC ROIs. Statistical values are reported in Supplementary Table S1. In the CER and PT, 1 and 3 out of respective 12 classification accuracies missed the level of significance at 4 mm sphere size. Hence, the 4 mm sphere size was excluded from further analyses in CER and PT.

Table 1 shows the results of the 3-way rmANOVAs with the factors FAMILIARITY (familiar, unfamiliar partner actions), HEMISPHERE (left, right), and SIZE (4 mm [only for M1 and PMC], 6 mm, 8 mm sphere radius), performed on relative accuracies, separately for each ROI. Figure 2 illustrates the results for the ROIs with 6 mm radius. Mean accuracy values for all ROIs can be found in Supplementary Table S1.

**Table 1:** ANOVA results in the 4 ROIs.

		M1			PMC		
	*df*	*F*	*p*	$\eta_{p}^{2}$	*F*	*p*	$\eta_{p}^{2}$
Familiarity	1.38	0.02	0.903	< 0.01	0.13	0.724	< 0.01
Hemisphere	1.38	**6.02**	**0.019**	**0.14**	**8.39**	**0.006**	**0.18**
Size	2.76	**36.82**	**< 0.001**	**0.49**	**39.03**	**< 0.001**	**0.51**
Fam. × Hem.	1.38	0.04	0.838	< 0.01	0.74	0.396	0.02
Fam. × Size	2.76	0.58	0.520	0.02	0.38	0.612	0.01
Hem. × Size	2.76	1.49	0.234	0.04	1.00	0.374	0.03
Fam. × Hem. × Size	2.76	0.70	0.457	0.02	0.11	0.893	< 0.01

		CER			PT		
	*df*	*F*	*p*	$\eta_{p}^{2}$	*F*	*p*	$\eta_{p}^{2}$
Familiarity	1.38	2.66	0.111	0.07	0.74	0.396	0.02
Hemisphere	1.38	0.47	0.496	0.01	0.20	0.654	0.01
Size	1.38	1.93	0.172	0.05	**11.22**	**0.002**	**0.23**
Fam. × Hem.	1.38	0.00	0.982	0.01	0.47	0.499	0.01
Fam. × Size	1.38	0.01	0.937	0.01	3.47	0.070	0.08
Hem. × Size	1.38	**4.42**	**0.042**	**0.10**	0.31	0.579	0.01
Fam. × Hem. × Size	1.38	0.00	0.994	0.01	0.74	0.396	0.02

Significant results are printed in bold font. M1, primary motor cortex; PMC, premotor cortex; CER, cerebellar lobule VIII; PT, planum temporale.

M1, PMC, and PT showed a main effect of sphere SIZE (all *p*s < 0.003), replicating generally increasing relative accuracies with growing ROI size reported in the literature (e.g., de Manzano et al., 2020). More interestingly, relative classification accuracies in M1 and PMC differed significantly between hemispheres irrespective of sphere size as indicated by main effects of HEMISPHERE in both regions (M1: *p* = 0.019; PMC: *p* = 0.006), in the absence of interactions involving HEMISPHERE and sphere SIZE (*p*s > 0.234). Most importantly, both ROIs showed effects with *opposite* lateralization: While mean accuracies in M1 were higher in the left than in the right hemisphere, the opposite was true in PMC, showing higher relative accuracies in the right than in the left hemisphere (see Figure 2 and Supplementary Table S1). These results are compatible with dissociated representations of self- (M1) and other-produced actions (PMC) related to the right and left hand, respectively.

As expected, accuracies in M1 did not differ depending on FAMILIARITY with the partner’s action (main effect of FAMILIARITY or interactions: *p*s > 0.457), in line with the idea that M1 represents self-produced actions (which were familiar for all pieces). However, unexpectedly, no effects of FAMILIARITY with the partner’s action were found in PMC either (*p*s > 0.396).

In CER and PT, relative accuracies did not differ, neither as a function of HEMISPHERE nor of FAMILIARITYA significant two-way interaction of HEMISPHERE × sphere SIZE in CER (*p* = 0.042) proved inconclusive when resolved with 2 paired *t*-tests comparing accuracies between the left and right hemisphere for each sphere SIZE [6 mm: *t* (77) = −1.460, *p* = 0.297; 8 mm: *t* (77) = 0.323, *p* = 0.748; FDR-corrected *p*-values].

### 3.2 Multivariate pattern analysis with whole-brain searchlight approach

The whole-brain searchlight analysis yielded no significant differences between classification accuracies for pieces with familiar and unfamiliar partner actions, mirroring the findings of the ROI analysis.

### 3.3 Control analysis

The present analysis focuses on the first phrase of the musical pieces during which pianists performed together at 120 bpm (Figure 1). The original paradigm of Kohler et al. (2023) contained an additional second phrase during which pianists had to either speed up or slow down to a tempo indicated at the beginning of each trial (see *Methods*). It has been shown that these impending tempo changes in the second phrase have subtle effects on performance timing already in the first phrase, indicative of pianists’ long-range planning (Kohler et al., 2023; see also Gugnowska et al., 2022; Novembre et al., 2016). To account for spurious effects of these anticipated tempo changes on the reported classification accuracies, we re-ran all ROI and searchlight analyses by adding the mean absolute asynchronies between partners’ keystrokes of the first phrase as a parametric modulator of no interest to the design matrix. The results of this control analysis (Supplementary Table S2) were highly similar to those described above, excluding that the present results were driven by the tempo change manipulation.

## 4 Discussion

The present study investigated neural processes underlying synchronous joint action in music performance by using multivariate pattern analysis (MVPA) to dissociate neural representations of self- and other-produced actions in auditory-motor regions of duetting pianists. We re-analyzed fMRI-scans from pianists performing melody-bassline duets with a partner, where we manipulated whether, prior to the experiment, pianists had previously rehearsed their own right-hand melody part only (unfamiliar bassline), or if they previously rehearsed both their right-hand part in addition to their partner’s left-hand part (familiar bassline) (Kohler et al., 2023). The data show higher accuracies in left M1 and right PMC. Based on previous studies, the most plausible interpretation of these findings is that pianists represented contents of their own right-hand action in left M1 concurrently with contents of their partner’s left-hand action in right PMC, as will be explained below. These simultaneous representations at different levels of the cortical motor hierarchy (reflecting execution of own and simulation of the partner’s action in M1 and PMC, respectively) lend initial evidence for parallel self and other internal forward models proposed by theories of joint action (Keller et al., 2016; Müller et al., 2021; Novembre and Keller, 2014; Pacherie, 2008; Wolpert et al., 2003). Future studies using novel tools like interbrain representational similarity analyses (Varlet and Grootswagers, 2024) may further substantiate this notion by demonstrating the representational alignment between partners’ M1 and PMC more directly. Interestingly, contents of familiar and unfamiliar partner actions were represented with similar precision. This seems to contrast previous findings showing global activity increases in motor regions when performing duets with familiar accompaniments (Kohler et al., 2023) or when observing familiar actions (Aglioti et al., 2008; Calvo-Merino et al., 2005, 2006; Candidi et al., 2014; Ticini et al., 2019). However, motor representations of unfamiliar accompaniments were likely generalized from the familiar accompaniments, based on the similarity of musical structures, potentially triggered by the external auditory perception of the partner’s part (Apšvalka et al., 2018; Pfordresher, 2012; see also de Manzano et al., 2020). Indeed, such a transfer is highly possible especially as our participants were highly trained pianists. Overall, findings across studies suggest that fine-grained activity patterns and global activity changes complement each other and elucidate how action contents are *represented* and *used* for simulating, anticipating, and coordinating one’s own and other’s actions during social interaction.

### 4.1 Lateralization suggests distinct representations of self- and other-produced actions

Classification accuracies were overall higher in left than right M1, i.e., in primary motor areas controlling the right hand used by the pianist to play the melodies. It is well established that M1 involvement is strongly lateralized towards the hemisphere contralateral to movement execution, reflected both in stronger activity (see, e.g., Chettouf et al., 2020; Horenstein et al., 2009) as well as higher classification accuracy (Kornysheva and Diedrichsen, 2014; Nambu et al., 2015; Wiestler and Diedrichsen, 2013). Accordingly, our results can be interpreted as suggesting that left M1 represented self-related information about the ongoing right-hand execution of the melody. Future studies could investigate in more detail how exactly pianists represent their own actions during joint music performance, as individual keypresses or chunked finger sequences, in terms of key-to-finger mappings, force profiles or rhythm and timing of keypresses (for studies starting to tackle these questions in individuals performing non-musical motor sequences; see Kornysheva and Diedrichsen, 2014; Yokoi et al., 2018; Yokoi and Diedrichsen 2019).

Lateralization was reversed in PMC, that is, classification accuracies were higher in the right than left hemisphere. This lateralization is interesting, not only because activity in PMC is typically less strongly lateralized than in M1, especially in complex sequential motor tasks and univariate analyses (for review, see Chettouf et al., 2020). Notably, multivariate studies that have reported (weakly) lateralized motor representations in PMC, clearly linked these representations to movements of the contralateral hand (e.g., Diedrichsen et al., 2013; Kornysheva and Diedrichsen, 2014; Wiestler and Diedrichsen, 2013). In the present study, this corresponds to the left hand, used by the partner. Additionally, MVPA studies on action *observation* have shown that PMC carries information related to contralateral hand actions performed by others (Errante et al., 2021; Filimon et al., 2015). For example, Errante et al. (2021) were able to decode from left PMC which type of grip participants observed in videos of a right hand opening or closing a box lid. Although these studies rarely compared classification accuracies between ipsi- and contralateral PMC, or sometimes reported bilateral representations (Apšvalka et al., 2018), these combined findings are compatible with the idea that the neural patterns we found in right PMC reflect representations of the contralateral left-hand actions performed by the partner.

However, before drawing any definite conclusions, several alternatives should be considered: For example, it might be argued that right PMC represents (i) *ipsilateral* hand actions, potentially merely mirroring the left-hemispheric patterns of *self*-produced movements, (ii) the *integration* of left- and right-hand actions in a bimanual task, rather than left-hand representations, or (iii) just trivially hand dominance. Yet, none of these alternatives can fully explain the stronger representations in right than left PMC: Interpretation (i) does not seem plausible as ipsilateral representations are usually weaker than their contralateral counterparts (for reviews, see Bundy and Leuthardt, 2019; Chettouf et al., 2020), while for interpretation (ii), bimanual integration has been shown bilaterally (e.g., Diedrichsen et al., 2013). Finally, interpretation (iii) is unlikely as right-hand dominance has been consistently shown to lateralize to left PMC (for review, see Goble and Brown, 2008). Hence, the most plausible interpretation for now remains that the information in right PMC reflects the representation of the contralateral left-hand basslines performed by the partner.

Furthermore, it might be argued that the literature underlying the present interpretation often concerns unimanual solo actions. However, the field is steadily scaling up to more complex (complementary) bimanual (e.g., Diedrichsen et al., 2013) or joint actions (Cirillo et al., 2018; Sacheli et al., 2022) and is beginning to reveal which mechanisms generalize to more ecologically valid motor behavior as tested here. Our approach may further contribute to that discussion by adding a solo and truly bimanual condition to the paradigm. Ultimately, strongest support for our conclusions may be gained by means of novel tools like interbrain representational similarity analyses (Varlet and Grootswagers, 2024) which provide a more direct way of measuring aligned representations between partners’ M1 (self) and PMC (other).

Another question is whether these representations pertain to the motor *simulation* of the partner’s part, or the *inhibition* of the corresponding left-hand movements. Arguments for the former interpretation can be derived from previous TMS studies using a similar duetting paradigm (e.g., Novembre et al., 2012). In these studies, pianists performing melodies with a partner who played the basslines showed increased (rather than decreased) excitability of right hand motor cortex, i.e., larger (rather than smaller) motor-evoked potentials related to the partner’s left-hand part. This pattern is incompatible with inhibition and supports the notion of simulation. It should be noted that activity patterns reminiscent of inhibition were also found, but only during solo performance of the melodies, not when pianists performed in duet with a partner (Novembre et al., 2012), as in the present study. Such inhibitory patterns may reflect the suppression of mirror movements in the contra-lateral hand (Bundy and Leuthardt, 2019; Chettouf et al., 2020; Welniarz et al., 2015). Overall, these combined results suggest that social interactive settings facilitate motor simulation rather than inhibition of partner actions, consistent with previous work (e.g., Sacheli et al., 2019) and reflected in the present right-lateralized PMC patterns.

Overall, the opposite lateralization in M1 and PMC suggests distinct representations of self and other at different levels of the cortical motor hierarchy: while the findings in M1 likely reflect the *execution* of self-produced right-hand melodies, the findings in right PMC likely reflect the *simulation* of partner-produced left-hand basslines, aligning with its role in motor simulation (Sacheli et al., 2019, 2022). This M1-PMC integration reveals an initial glimpse into how bimanual actions are coordinated simultaneously within a dyadic motor plan, where agents would apply sensorimotor control processes for both self and partner actions (Sacheli et al., 2021). It underscores the simultaneity and content-specificity of internal forward models for self- and other-produced actions, predicted by theories of joint performance coordination (Keller et al., 2016; Müller et al., 2021; Sebanz and Knoblich, 2009).

### 4.2 Auditory-motor transfer of other-produced actions

Another strategy that we employed to identify neural representations of other-produced actions was by manipulating motor familiarity. We hypothesized that compared to being unfamiliar with a co-performer’s accompanying part in a duet (i.e., the bassline), familiarity with the other’s part would evoke stronger internal modelling, that is, stronger motor (in PMC and CER) and possibly also auditory (in PT) representations (Jeannerod, 2006; Keller et al., 2016; Kohler et al., 2023; Müller et al., 2021; Novembre and Keller, 2014; Patel and Iversen, 2014), reflected by increased classification accuracy. However, there was no significant difference in classification accuracy between familiar and unfamiliar conditions in any brain area. While the absence of effects in CER and PT may be explained, e.g., by overall higher noise levels in cerebellar than cerebral cortical fMRI signals (Kornysheva and Diedrichsen, 2014; Wiestler et al., 2014), and a saturation of PT activity due to the ongoing perception of the jointly performed pieces (de Manzano et al., 2020; May et al., 2022; Regev et al., 2021; Schindler et al., 2013), the PMC findings deserve more in depth discussion.

One possible explanation for the non-significant effect of familiarity in (right) PMC is auditory-motor transfer, that is, the emergence of motor patterns from the auditory perception of the basslines. In expert pianists, such as our participants, auditory and motor systems are strongly coupled (Bangert et al., 2006; Baumann et al., 2007; Jäncke, 2012; Novembre and Keller, 2014; Zatorre et al., 2007). Therefore, simply hearing the bassline (performed by the partner) may have indeed evoked bottom-up auditory-informed motor patterns in PMC, even when the basslines were unfamiliar. This effect may have been reinforced by the ability of pianists to generalize motor patterns across similar sequences, based on their long-term musical training (Meyer and Palmer, 2003; Palmer and Meyer, 2000; Pfordresher, 2012). In the current study, all stimuli were simple Bach chorale sequences that were repeated several times during a session, making it possible that the expert pianists in our study generalized across familiar and unfamiliar accompaniments, based on common abstract structural characteristics. This idea finds general support in two recent MVPA studies in non-pianists who exhibited comparable classification accuracies in motor areas for trained and untrained finger sequences with similar structure, after 4 sessions of observational training (Apšvalka et al., 2018), or even just only 20 min of piano training, compared to novices (de Manzano et al., 2020). This demonstrates that content-specific neural motor representations can generalize across similar pieces when passively watching or listening to another piece, an effect that may have been particularly strong in our highly trained participants (see *Methods*). Together, the high classification accuracy irrespective of familiarity may derive from the bottom-up/top-down interplay in auditory-motor systems. In pianists with long-term musical knowledge, hearing the basslines may have evoked bottom-up audio-informed representations in PMC (de Manzano et al., 2020), which might be indistinguishable from top-down motor-informed representations that generalize across structurally similar sequences.

A final, broader conceptual consideration that should be highlighted here is the complementarity of insights that can be gained from univariate and multivariate analyses. Contrary to the present findings, univariate analyses yielded significant effects of familiarity, that is, increased activity and connectivity in familiar conditions (Kohler et al., 2023), revealing the potential *use* of motor knowledge for simulating partner actions. In contrast, MVPA (the current study) reveals the *representation* of motor patterns, irrespective of whether they are more motor- or audio-informed. In other words, these findings based on either global activity changes (univariate analyses) or fine-grained activity patterns (multivariate analyses) may capture different aspects of neural processing: the use versus the representation of action content. Together, both approaches draw a more complete picture of the mechanisms of joint action, emphasizing the integration of self- and other-produced movements within cortico-cerebellar auditory-motor networks. This integration ultimately contributes to the dynamic embodiment required for smooth coordination in musical ensemble performance and, possibly, other forms of social interaction.

## 5 Conclusion

The current study provides initial evidence for parallel, distinct and content-specific auditory-motor representations of complementary self- and other-produced actions in musical duets. This was reflected in the opposite hemispheric lateralization of neural information concurrently represented in M1 (own-action execution) and PMC (simulation of partner actions), which cannot be explained by lateralization properties currently known from the motor literature. These results are in line with theories proposing distinct yet integrated self and other internal forward models contributing to smooth coordination in social interactions (e.g., Keller et al., 2016; Knoblich and Sebanz, 2006; Müller et al., 2021; Novembre and Keller, 2014). Notably, the precision of these representations was less dependent on motor familiarity than previously believed, suggesting that general auditory-motor piano practice, even without in-depth motor knowledge of a partner’s part, may lead to informed (forward) models that can support joint music performance. This extends the role of internal models beyond highly specific instances of motor familiarity. Future studies testing the representational alignment between partners’ motor systems more directly (Varlet and Grootswagers, 2024) should substantiate our conclusions and clarify to what extent our findings generalize to less experienced musicians and other forms of social interaction. More generally, this research highlights new ways of how to combine the complementary strengths of uni- and multivariate approaches to gain novel insights into the neural mechanisms underpinning human social actions.

## Data Availability

The datasets presented in this article are not readily available because of the lack of explicit consent from participants. Requests to access the datasets should be directed to daniela.sammler@ae.mpg.de.
