# Supplementary material for: Distinct and content-specific neural representations of self- and other-produced actions in joint piano performance
Source: Front Hum Neurosci. 2025 Mar 12;19:1543131. doi: 10.3389/fnhum.2025.1543131 (PMC11936940; doi:10.3389/fnhum.2025.1543131)
Supplement: Supplementary file 1 [file Data_Sheet_1.PDF]

Distinct and content-specific neural representations of self- and other-produced actions in joint piano performance

Natalie Kohler▲[1,2], Anna M. Czepiel▲[3,4], Örjan de Manzano[5,6], Giacomo Novembre[7], Peter E. Keller[8,9], Arno Villringer[1], Daniela Sammler*[2,10]

[1] Department of Neurology, Max Planck Institute for Human Cognitive and Brain Sciences, Leipzig, Germany

[2] Research Group Neurocognition of Music and Language, Max Planck Institute for Empirical Aesthetics, Frankfurt am Main, Germany

[3] Department of Music, Max Planck Institute for Empirical Aesthetics, Frankfurt am Main, Germany

[4] Department of Psychology, University of Toronto Mississauga, Mississauga, Ontario, Canada

[5] Department of Cognitive Neuropsychology, Max Planck Institute for Empirical Aesthetics, Frankfurt am Main, Germany

[6] Department of Neuroscience, Karolinska Institutet, Stockholm, Sweden

[7] Neuroscience of Perception and Action Laboratory, Italian Institute of Technology, Rome, Italy,

[8] Center for Music in the Brain, Department of Clinical Medicine, Aarhus University, Aarhus, Denmark,

[9] The MARCS Institute for Brain, Behaviour and Development, Western Sydney University, Australia,

[10] Department of Neuropsychology, Max Planck Institute for Human Cognitive and Brain Sciences, Leipzig, Germany

***Correspondence:**
Corresponding Author
daniela.sammler@ae.mpg.de

▲Authors contributed equally.

Keywords: Joint action, fMRI, MVPA, music performance, pianists, motor simulation, internal models.

frontiers

Supplementary material

Table S1. Mean and *SD* of classification accuracies and related chance-levels in the ROI analysis.

		left M1						right M1					
		Accuracy		Chance		Difference		Accuracy		Chance		Difference	
	Radius	Mean	*SD*	Mean	*SD*	*T*	*p*	Mean	*SD*	Mean	*SD*	*T*	*p*
F1 vs. F2	4	0.63	.117	0.50	.001	6.94	< .001	0.57	.119	0.50	.001	3.45	.001
	6	0.66	.121	0.50	.001	8.33	< .001	0.61	.106	0.50	.001	6.62	< .001
	8	0.67	.141	0.50	.001	7.64	< .001	0.65	.121	0.50	.001	7.90	< .001
U1 vs. U2	4	0.62	.142	0.50	.001	5.12	< .001	0.58	.117	0.50	.001	4.28	< .001
	6	0.66	.147	0.50	.001	6.64	< .001	0.61	.120	0.50	.001	5.62	< .001
	8	0.69	.154	0.50	.001	7.64	< .001	0.66	.134	0.50	.001	7.40	< .001

		left PMC						right PMC					
		Accuracy		Chance		Difference		Accuracy		Chance		Difference	
	Radius	Mean	*SD*	Mean	*SD*	*T*	*p*	Mean	*SD*	Mean	*SD*	*T*	*p*
F1 vs. F2	4	0.58	.112	0.50	.001	4.19	< .001	0.62	.131	0.50	.001	5.62	< .001
	6	0.61	.131	0.50	.001	5.07	< .001	0.67	.141	0.50	.001	7.61	< .001
	8	0.64	.136	0.50	.001	6.56	< .001	0.69	.139	0.50	.001	8.63	< .001
U1 vs. U2	4	0.57	.110	0.50	.001	3.78	< .001	0.59	.134	0.50	.001	4.22	< .001
	6	0.61	.135	0.50	.001	5.13	< .001	0.65	.167	0.50	.002	5.64	< .001
	8	0.66	.134	0.50	.001	7.31	< .001	0.68	.186	0.50	.002	5.90	< .001

		left CER						right CER					
		Accuracy		Chance		Difference		Accuracy		Chance		Difference	
	Radius	Mean	*SD*	Mean	*SD*	*T*	*p*	Mean	*SD*	Mean	*SD*	*T*	*p*
F1 vs. F2	4	0.57	.103	0.50	.001	4.16	< .001	**0.52**	**.121**	**0.50**	**.001**	**1.25**	**.111**
	6	0.63	.098	0.50	.001	8.50	< .001	0.61	.105	0.50	.001	6.32	.001
	8	0.63	.081	0.50	.001	9.77	< .001	0.63	.126	0.50	.001	6.47	< .001
U1 vs. U2	4	0.57	.110	0.50	.001	3.69	< .001	0.57	.124	0.50	.001	3.38	< .001
	6	0.60	.106	0.50	.001	6.11	< .001	0.58	.129	0.50	.001	3.81	< .001
	8	0.60	.100	0.50	.001	6.16	< .001	0.60	.127	0.50	.001	5.10	< .001

		left PT						right PT					
		Accuracy		Chance		Difference		Accuracy		Chance		Difference	
	Radius	Mean	*SD*	Mean	*SD*	*T*	*p*	Mean	*SD*	Mean	*SD*	*T*	*p*
F1 vs. F2	4	**0.51**	**.108**	**0.50**	**.001**	**0.84**	**.204**	0.54	.124	0.50	.001	2.10	.023
	6	0.55	.119	0.50	.001	2.83	.004	0.56	.153	0.50	.001	2.56	.008
	8	0.57	.120	0.50	.001	3.68	< .001	0.57	.146	0.50	.001	2.78	.005
U1 vs. U2	4	0.52	.120	0.50	.001	**1.26**	**.111**	**0.52**	**.107**	**0.50**	**.001**	**1.40**	**.090**
	6	0.58	.151	0.50	.001	3.45	.001	0.55	.106	0.50	.001	3.10	.002
	8	0.60	.155	0.50	.001	3.90	< .001	0.59	.117	0.50	.001	5.06	< .001

F1 vs. F2 refers to the classification of pieces with familiar partner actions, U1 vs. U2 to the classification of pieces with unfamiliar partner actions (M1, primary motor cortex; PMC, premotor cortex; CER, cerebellar lobule VIII; PT, planum temporale). Radius is shown in millimeters. Chance refers to the estimated chance level based on 10,000 permutations. Difference: Statistical difference between accuracy and chance. Non-significant values after FDR-correction are marked in bold at $\alpha = 0.05$.

Table S2. ANOVA results for separate ROIs in the control analysis.

		M1			PMC		
	df	*F*	*p*	n_p^2	*F*	*p*	n_p^2
Familiarity	1, 38	0.00	.947	< 0.01	0.10	.752	< 0.01
Hemisphere	1, 38	**4.19**	**.048**	**0.10**	**8.88**	**.005**	**0.19**
Size	2, 76	**41.22**	**< .001**	**0.52**	**45.22**	**< .001**	**0.54**
Fam. × Hem.	1, 38	0.04	.851	< 0.01	0.73	.399	0.02
Fam. × Size	2, 76	0.50	.552	0.01	1.03	.340	0.03
Hem. × Size	2, 76	1.19	.295	0.03	0.54	.584	0.01
Fam. × Hem. × Size	2, 76	0.23	.713	0.01	0.02	.971	<0.01
		CER			PT		
	df	*F*	*p*	n_p^2	*F*	*p*	n_p^2
Familiarity	1, 38	2.24	.143	0.06	2.17	.149	0.05
Hemisphere	1, 38	0.56	.461	0.01	0.04	.834	< 0.01
Size	2, 76	2.13	.153	0.05	**8.56**	**.006**	**0.18**
Fam. × Hem.	1, 38	0.01	.906	< 0.01	0.05	.828	< 0.01
Fam. × Size	2, 76	0.12	.734	< 0.01	0.66	.422	0.02
Hem. × Size	2, 76	**5.03**	**.031**	**0.12**	2.11	.154	0.05
Fam. × Hem. × Size	2, 76	0.05	.821	< 0.01	0.75	.392	0.02

Results of three-way ANOVAs with factors FAMILIARITY (Fam.; familiar, unfamiliar), HEMISPHERE (Hem.; left, right), and SIZE (either 4 mm, 6 mm, 8 mm for M1 and PMC, or 6 mm, 8 mm sphere radius for CER and PT) in the control analysis. Relative classification accuracies of pieces with familiar (F1, F2) and unfamiliar (U1, U2) partner actions were used as dependent variables in all ROIs (M1, primary motor area; PMC, premotor cortex; CER: cerebellar lobule VIII; PT, planum temporale). Significant *p*-values are shown in bold at $\alpha = 0.05$. Results were qualitatively highly similar to the original analysis, except for the absence of a main effect of SIZE in CER (p = .153). Similarly to the main analysis, the data show an interaction of HEMISPHERE × SIZE in CER (p = .031), followed by paired *t*-tests showing no significant differences between accuracies of the left and right hemispheres (6 mm: p = .260, 8 mm: p = .725, after FDR-correction).
